# Efficacy of the sam program in reducing suicidal ideation in high-risk adolescents: initial results from a randomized controlled trial

**DOI:** 10.1192/j.eurpsy.2026.10682

**Published:** 2026-07-31

**Authors:** W. A. Ahmed, B. G. Fernández, V. F. Rodrigues, A. D. L. T. Luque, M. D. Marsa

**Affiliations:** 1 Complutense University; 2 CIBERSAM; 3 Hospital Clinico San Carlos, Madrid, Spain

## Abstract

**Introduction:**

Suicide is one of the leading causes of death among adolescents. The high prevalence of suicidal ideation in this population highlights the need for brief and effective interventions. The *Self-Awareness of Mental Health*(SAM) program is a short therapeutic intervention focused on risk/protective factors and emotional coping skills.

**Objectives:**

This study aims to assess the efficacy of the SAM program in reducing suicidal ideation and to examine its effect in preventing suicidal behaviours among high-risk adolescents.

**Methods:**

A randomized controlled trial was conducted with 85 adolescents (aged 12–18) with high suicidal ideation, the sample was divided into two groups: 42 participants (clinical group) and 43 participants (control group). Participants were allocated to either an experimental group (SAM + standard care) or a control group (standard care only). Validated clinical tools were used: C-SSRS, SSI, among others. Pre- and post-intervention data were analyzed using repeated-measures ANOVA.

**Results:**

The SAM group showed a significant reduction in suicidal ideation intensity (C-SSRS) with a significant group–time interaction *(F(1,29) = 10.116, p = 0.003),* while the control group showed increased intensity (See Figure 1). The total scores on the SSI scale also decreased significantly in the SAM group *(F(1,35) = 8.41, p = 0.006), *with overall lower post-treatment scores *(F(1,35) = 5.514, p = 0.025).*

Both groups showed a decrease in the number of suicide behaviours (*p < 0.001*) and frequency (*p < 0.001*), but no significant between-group differences were found (*p > 0.5*).

**Image 1:**
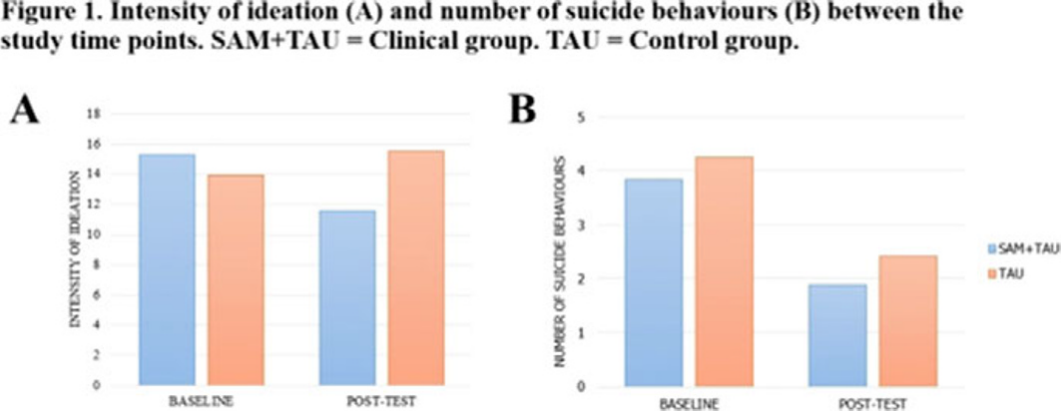
Image 1: Long description.

**Conclusions:**

The SAM program was effective in reducing suicidal ideation in high-risk adolescents, particularly in terms of intensity and intent. Its brief format and compatibility with standard treatment make it a practical and clinically applicable tool for suicide prevention in youth populations.

**Disclosure of Interest:**

None Declared

## Long descriptions

### Image 1: Long description

Panel A is a bar chart titled Intensity of Ideation. The y-axis ranges from 0 to 18. The x-axis shows two time points: Baseline and Post-test. At Baseline, the S A M plus T A U group (blue bar) is at approximately 15.3 and the T A U group (orange bar) is at 14. At Post-test, the S A M plus T A U group decreases to approximately 11.5, while the T A U group increases to approximately 15.5.

Panel B is a bar chart titled Number of Suicide Behaviours. The y-axis ranges from 0 to 5. The x-axis shows Baseline and Post-test. At Baseline, the S A M plus T A U group is at approximately 3.8 and the T A U group is at 4.3. At Post-test, both groups show a decrease: the S A M plus T A U group drops to approximately 1.9 and the T A U group drops to approximately 2.4.

A legend to the right of Panel B indicates that blue bars represent S A M plus T A U and orange bars represent T A U.

Navigate back to Image 1:.

